# Assessment of Community Interventions for Bystander Cardiopulmonary Resuscitation in Out-of-Hospital Cardiac Arrest: A Systematic Review and Meta-analysis

**DOI:** 10.1001/jamanetworkopen.2020.9256

**Published:** 2020-07-01

**Authors:** Yang Yu, Qingtao Meng, Sonali Munot, Tu N. Nguyen, Julie Redfern, Clara K. Chow

**Affiliations:** 1 Department of Emergency, Xinhua Hospital Affiliated to Shanghai Jiao Tong University School of Medicine, China; 2 Westmead Applied Research Centre, Faculty of Medicine and Health, University of Sydney, Sydney, New South Wales, Australia; 3 Department of Cardiology, West China Hospital of Sichuan University, China; 4 The George Institute for Global Health, UNSW, Sydney, New South Wales, Australia; 5 Department of Cardiology, Westmead Hospital, Sydney, New South Wales, Australia

## Abstract

**Importance:**

Outcomes from out-of-hospital cardiac arrests (OHCAs) remain poor. Outcomes associated with community interventions that address bystander cardiopulmonary resuscitation (CPR) remain unclear and need further study.

**Objective:**

To examine community interventions and their association with bystander CPR and survival after OHCA.

**Data Sources:**

Literature search of the MEDLINE, Embase, and the Cochrane Library databases from database inception to December 31, 2018, was conducted. Key search terms included *cardiopulmonary resuscitation*, *layperson*, *basic life support*, *education*, *cardiac arrest*, and *survival*.

**Study Selection:**

Community intervention studies that reported on comparisons with control and differences in survival following OHCA were included. Studies that focused only on in-hospital interventions, patients with in-hospital cardiac arrest, only dispatcher-assisted CPR, or provision of automated external defibrillators were excluded.

**Data Extraction and Synthesis:**

Pooled odds ratios (ORs) and 95% CIs were estimated using a random-effects model. This study followed the Preferred Reporting Items for Systematic Reviews and Meta-analyses (PRISMA) reporting guideline.

**Main Outcomes and Measures:**

Thirty-day survival or survival to hospital discharge and bystander CPR rate.

**Results:**

A total of 4480 articles were identified; of these, 15 studies were included for analysis. There were broadly 2 types of interventions: community intervention alone (5 studies) and community intervention combined with changes in health services (10 studies). Four studies involved notification systems that alerted trained lay bystanders to the location of the OHCA in addition to CPR skills training. Meta-analysis of 9 studies including 21 266 patients with OHCA found that community interventions were associated with increased survival to discharge or 30-day survival (OR, 1.34; 95% CI, 1.14-1.57; *I*^2^ = 33%) and greater bystander CPR rate (OR, 1.28; 95% CI, 1.06-1.54; *I*^2^ = 82%). Compared with community intervention alone, community plus health service intervention was associated with a greater bystander CPR rate compared with community alone (community plus intervention: OR, 1.74; 95% CI, 1.26-2.40 vs community alone: OR, 1.06; 95% CI, 0.85-1.31) (*P* = .01). Survival rate, however, was not significantly different between intervention types: community plus health service intervention OR, 1.71; 95% CI, 1.09-2.68 vs community only OR, 1.26; 95% CI, 1.05-1.50 (*P* = .21).

**Conclusions and Relevance:**

In this study, while the evidence base is limited, community-based interventions with a focus on improving bystander CPR appeared to be associated with improved survival following OHCA. Further evaluations in diverse settings are needed to enable widespread implementation of such interventions.

## Key Points

**Question:**

Are community interventions aimed to improve bystander cardiopulmonary resuscitation associated with outcomes of out-of-hospital cardiac arrest in the communities that received interventions?

**Findings:**

This systematic review and meta-analysis of 9 studies including 21 266 out-of-hospital cardiac arrests found that community interventions were associated with better out-of-hospital cardiac arrest survival and bystander cardiopulmonary resuscitation rates; the difference for both outcomes was approximately 1.3-fold with vs without community interventions. Despite moderate to high interstudy heterogeneity, sensitivity analyses supported the main result.

**Meaning:**

The results of this study suggest that community interventions may be associated with better rates of bystander cardiopulmonary resuscitation and patient survival after out-of-hospital cardiac arrest.

## Teaser

This systematic review and meta-analysis examines the rates of bystander cardiopulmonary resuscitation and use of interventions used for training.

## Introduction

Out-of-hospital cardiac arrest (OHCA) is the cessation of cardiac mechanical activity and the absence of signs of circulation that happens outside of the hospital setting. Out-of-hospital cardiac arrest is a challenging global public health issue, and the estimated incidence of OHCA treated and recorded after emergency medical service (EMS) intervention ranges from 14.9 to 110.8 per 100 000 persons worldwide.^1,2,3^ Despite awareness of this issue, the average survival rate following OHCA remains poor, at approximately 10%, with little improvement in recent decades.^1,2,3,4^ The pathway for improving survival includes a set of sequentially resuscitative interventions conceptualized as the chain of survival.^5^

Early recognition and initiation of cardiopulmonary resuscitation (CPR) by bystanders are key links in this chain and, in observational studies, have been associated with a 2- to 4-fold increase in survival and favorable outcomes.^6,7^ In the past decade, various innovative initiatives and interventions have been implemented in many nations and regions to improve bystander CPR rates, including the Take Heart America program,^8^ HeartRescue Project,^9^ TAKE10 program,^10^ Lifesavers campaign in England,^11^ and World Restart a Heart initiative.^12^ Also in the past decade, novel approaches and technologies have been introduced to facilitate the learning of CPR skills among laypeople, such as hands-only CPR, brief video kits, mobile applications, or social media broadcasting.^13,14^

Increases in a community’s training and engagement with CPR have also been reported to be associated with improved survival rates following OHCA.^8,15^ A recent review highlighted the efficacy of interventions conducted by health services to improve CPR,^16^ such as dispatcher-assisted CPR. Yet, while education and training of the lay public in CPR and basic life support have been well recognized and taught since the early 1970s,^17^ there is little quantification of the potential results of community interventions, which often involve training the lay public in CPR, in improving bystander CPR.

To address this issue, we conducted a systematic review and meta-analysis of studies that included intervention and control comparisons to evaluate the outcomes of community-based programs aimed at improving bystander CPR associated with rates of bystander CPR and survival following OHCA.

## Methods

### Search Strategy

This study followed the Preferred Reporting Items for Systematic Reviews and Meta-analyses (PRISMA) reporting guideline.^18^ Searches for relevant publications were conducted in the following databases from database inception until December 31, 2018: Ovid MEDLINE (from 1946), Embase (from 1980), and Cochrane Central Register of Controlled Trials. We also searched the reference lists of articles reporting eligible studies and relevant reviews for additional published data. Key words and Medical Subject Headings terms included *cardiopulmonary resuscitation*, *layperson*, *basic life support*, *education*, *cardiac arrest*, and *survival*. The full electronic search strategy can be seen in the eMethods in the Supplement.

We established the eligibility criteria to address our research question following the PICO (population, intervention, comparison, outcome) format. The population included patients with OHCA. Interventions comprised community intervention programs aimed to improve bystander CPR and survival following OHCA. Community interventions were defined as interventional programs that included community-based intervention alone or community intervention combined with changes in health services. We examined studies on community-based interventions compared with no interventions. Outcomes included survival to hospital discharge or 30 days and bystander CPR. Published original research articles were included if they reported randomized, nonrandomized interventional, or observational studies.

Only articles published in English were included. Studies in children, animal studies, letters, case reports, abstracts, conference papers, commentaries and editorials, reviews, studies that did not present original data, studies including in-hospital cardiac arrest, or those that did not report survival rates after OHCA were excluded. Studies that reported only dispatcher-assisted CPR or automated external defibrillators without a community intervention component were also excluded.

The primary outcomes of interest in the studies analyzed were 30-day survival or survival to hospital discharge of OHCA and rate of bystander CPR for OHCA. Other outcomes reported by the studies were also extracted, including proportion of bystander-witnessed cardiac arrests, proportion of automated external defibrillator use by bystanders, return of spontaneous circulation, survival to arrival at hospital, and neurologic outcomes.

Titles and abstracts were screened by 2 of us (Y.Y. and Q.M.) to identify eligible studies in accordance with the inclusion criteria. Full-text articles of the selected studies were then independently appraised by 2 of us (Y.Y. and S.M.) and disagreement was resolved by discussion until consensus was reached. In cases in which there were research studies with multiple publications of the results, we used the more recent or complete publication. If patient data overlapped between publications, those studies were considered as duplicates. To ensure capture of all related studies, all reference lists of the screened full-text studies were visually scanned for additional articles not found through the search strategy.

Data were extracted into a predetermined table based on recommendations in the Cochrane Handbook for Systematic Reviews.^19^ The extracted data were validated by 2 of us (Y.Y. and S.M.) and any discrepancies were resolved via discussion. Major categories in the data extraction table included authors; title; publication year; study period, location, and design; targeted population; number of OHCAs; type of interventions; and outcomes (survival to discharge, 30-day survival, and bystander CPR). Data on other outcomes reported by the studies are presented in eTable 1 in the Supplement.

Risk of bias for randomized clinical trials was assessed using the Cochrane Collaborations^19^ tool for assessing risk of bias, and the Newcastle-Ottawa Scale^20^ was used to assess the risk of bias for nonrandomized interventional studies and observational studies. The Newcastle-Ottawa Scale allocates stars for quality of 3 components (selection of cases, comparability of cohorts, and assessment of outcome). A study can be assigned 0 to 9 stars, with 9 stars representing a low risk of bias and 0 stars indicating a high risk of bias. Disagreements on quality assessment were resolved through consultation with one of us (C.K.C.).

### Statistical Analysis

Review Manager (RevMan), version 5.3 (The Cochrane Collaboration, 2014), was used to perform meta-analysis of the study data. We pooled study data using a random-effects model with sensitivity analyses owing to the anticipated significant heterogeneity between studies. The random-effects model is the most conservative approach in this setting because it incorporates between-study heterogeneity. Outcomes are reported as OR and 95% CI as a relative measure of association. Statistical heterogeneity across the studies was measured by the χ^2^ test and quantified with the *I*^2^ statistic. The *I*^2^ value of 25% or less represent low inconsistency; 50%, moderate inconsistency; and 75% or more, high inconsistency. Sensitivity analyses were performed to explore the role of a single study in the overall pooled estimate by omitting one study at a time. Subgroup differences were examined by χ^2^ analysis, and a 2-tailed, unpaired *P* value <.05 was considered statistically significant.

## Results

### Study Selection and Characteristics

Through the initial literature search, we identified 4480 records. After the removal of duplicates, the remaining 2271 studies were assessed for inclusion through title and abstract screening. Of these, 89 studies were reviewed for full-text eligibility and a further 74 were excluded, leaving 15 studies that met our inclusion criteria (eFigure 1 in the Supplement).

The final 15 studies reported a median study duration of 36 months (range, 12-126 months). Only 1 study was a randomized clinical trial, 3 studies were nonrandomized controlled trials, and the others were either prospective (n = 4) or retrospective (n = 7) observational studies that included a control comparison. Among these 15 studies, 6 were from the US, 2 were from Sweden, 2 were from Denmark, and the other 5 studies were from the Netherlands, Singapore, Korea, Japan, and Australia. Ten studies used data from cardiac arrest registries and 5 studies obtained data from an EMS dispatcher center or hospital medical records. In terms of outcomes, 11 studies reported survival rate to hospital discharge, whereas the remaining reported an outcome of 30-day survival. All studies reported changes in bystander CPR rates. The characteristics and quality assessment of the included studies are summarized in the Table.

**Table.** Characteristics of Studies Included

Source	Location	Study period	Populations for training	Study design	No. of people with OHCA	Survival to discharge/30-d survival	Bystander CPR rate	Newcastle-Ottawa Scale Score^a^
Smith et al,^21^ 2001	Australia	1998-1999	Fire officers at 7 fire stations in intervention area	Comparison made between the populations where the intervention took place and the populations where there was no intervention	Control: 268; intervention: 161	Control: 4%; intervention: 4%	Control: 25%; intervention: 20%	7
Lick et al,^8^ 2011	US	2004-2009	School students and their families, city employees	Comparison made in the same population before and after intervention	Control: 106; intervention: 247	Control: 8.5%; intervention: 19%; (OR, 2.6; 95% CI, 1.19-6.26); *P* = .011	Control: 20%; intervention: 29% (OR, 1.7; 95% CI, 0.96-2.89; *P* = .086	6
Nielsen et al,^22^ 2014	Denmark	2008-2010	General population	Comparison made in the same population before and after intervention	Control: Danish cardiac arrest registry, 2001-2003 (No. not provided); intervention: 96	Control: 0; intervention: 5.4	Control: 22; intervention: 47; *P* = .001	6
Ringh et al,^23^ 2015	Sweden	2012 -2013	9828 laypeople from the general population trained	Randomized clinical trial	Control: 361; intervention: 306	Control: 8.6; intervention: 11.2; (absolute difference, 2.6; 95% CI, −2.1 to 7.8); *P* = .28	Control: 48	NA
							; intervention: 62; (absolute difference, 14; 95% CI, 6-21); *P* < .001	
Ro et al,^24^ 2015	Japan, Korea	2006-2011	General population	Comparison made in the same population before and after intervention	Control: 4613; intervention: 7048	Control: 7.1; intervention: 8.6 (OR, 1.24; 95% CI, 1.07-1.42)	Control: 25.9; intervention: 35.0 (OR, 1.54; 95% CI, 1.42-1.67)	6
Pijls et al,^25^ 2016	The Netherlands	2012-2014	Trained volunteers in the community	Comparison made between the group where the intervention took place and the group where there was no intervention	Control: 131; intervention: 291	Control: 16; intervention: 27.1 (OR, 1.95; 95% CI, 1.1-3.33); *P* = .014	Control: 65.3; intervention: 61.5; *P* < .001	7
Hasselqvist-Axe et al,^26^ 2017	Sweden	2012-2014	Firefighters and police officers	Comparison made between populations where the intervention took place and populations where there was no intervention	Control: 5155; intervention: 3543	Control: 7.7; intervention: 9.5 (OR, 1.27; 95% CI, 1.05-1.54)	Control: 58.3; intervention: 59.2	6
Hwang et al,^27^ 2017	Korea	2009-2013	General population	Comparison made in the same population before and after intervention	Control: 182; intervention: 282	Control: 8.8; intervention: 18.1; *P* < .05	Control: 15.9; intervention: 50.4; *P* < .001	6
Uber et al,^28^ 2018	US	2010-2015	Nontargeted, passersby at 7 public locations	Comparison made in the same population before and after intervention	Control: 899; intervention: 587	Control: 10; intervention: 10; (β, −0.02; 95% CI, −0.11 to 0.06); *P* = .98	Control: 37; intervention: 36 (β, −0.002; 95% CI, −0.16 to 0.15); *P* = .77	5
Wissenberg et al,^29^ 2013	Denmark	2001-2010	Elementary school pupils, drivers	Retrospective, observational	2001: 1262; 2010: 1906	2001: 3.5; 2010: 10.8; *P* < .001; no OR provided	2001: 21.1; 2010: 44.9; *P* < .001; no OR provided	5
Malta Hansen et al,^30^ 2015	US	2010-2013	General population	Retrospective, observational	2010: 1167; 2013: 1341	2010: 7.1; 2013: 9.7; *P* = .02; no OR provided	2010: 39.3; 2013: 49.4; *P* < .01; no OR provided	5
Lai et al,^31^ 2015	Singapore	2001-2012	General population	Retrospective, observational	Before: 2428; after: 3025	Before: 1.6; after: 3.2 (OR, 2.2; 95% CI, 1.5-3.3)	Before: 19.7; after: 22.4; *P* = .02; no OR provided	5
van Diepen et al,^32^2017	US	2011-2015	General population	Retrospective, observational	2011: 6762; 2015: 16 103	2011: 13.7; 2015: 10.5; *P* < .001; no OR provided	2011: 41.8; 2015: 43.5; *P* < .001; no OR provided.	5
Fordyce et al,^15^ 2017	US	2010-2014	General population	Retrospective, observational	Home,2010: 1063; 2014: 1242; public, 2010: 470; 2014: 605	Home, 2010: 5.7; 2014: 8.1; *P* = .047; public, 2010: 10.82014: 16.2; *P* = .04; no OR provided	Home, 2010: 28.3; 2014: 41.3; *P* < .001; public, 2010: 61; 2014: 70.5; *P* = .01; no OR provided	5
Adabag et al,^33^ 2017	US	2011-2014	General population	Retrospective, observational	2011: 1067; 2014:1473	2011: 16; 2014:12; *P* = .01; no OR provided	2011: 26; 2014: 38; *P* < .0001; no OR provided	5

Abbreviations: CPR, cardiopulmonary resuscitation; OHCA, out-of-hospital cardiac arrest; NA, not applicable; OR, odds ratio.

^a^ The Newcastle- Ottawa Scale allocates stars for quality of 3 components (selection of cases, comparability of cohorts, and assessment of outcome); a score can range from 0 to 9 stars, with 9 stars representing a low risk of bias, and 0 stars indicating a high risk of bias.

### Interventions

There were broadly 2 types of interventions: community intervention alone and community intervention combined with changes in health service. In the studies included in this review, community-level interventions included public CPR skills training (standard basic life support courses or compression-only CPR), distribution of self-instruction CPR kits to public schools or school students, broadcasting resuscitation training on television or other media, mandatory CPR training for school students, when acquiring a driver's license or for some occupations (eg, firefighters, policemen, and rescue squads), and messaging trained laypersons or first responders to encourage attendance at cardiac arrest sites. Program components at the level of health systems included strengthening of EMS systems and implementing advanced life support protocols in hospitals, increasing numbers of ambulances, and training of EMS and hospital staff in high-performance CPR skills, early emergency cardiac catheterization, and use of therapeutic hypothermia. Details of these interventions are presented in eTable 2 in the Supplement.

Community interventions alone were reported in 5 studies, while in the remaining 10 studies, comprehensive interventions were launched with both community-training and health service components. Among the 5 studies with community-only interventions, 1 study reported a single point-of-contact, compression-only CPR training session for passersby at public locations,^28^ while the other 4 studies reported the use of notification systems, such as text messages, in addition to CPR skills training.^21,23,25,26^ Among the 10 studies with combined community and health services intervention, training or retraining of EMS and hospital staffs was reported in 8 studies,^8,15,22,24,27,29,30,31^ and improving therapeutic hypothermia and revascularization was the focus in 4 studies.^8,29,31,33^ In the Minnesota Resuscitation Consortium,^33^ there was an innovation in the organization structure in which first responders, EMS, police and fire departments, hospital emergency departments, and cardiology, intensive care unit, neurology, and physical therapy/rehabilitation services were gathered under the same organization. Ten studies described interventions in enough detail to be easily followed or replicated.^8,21,22,23,24,25,26,27,28,29^

### Meta-analyses

**Figure 1.**
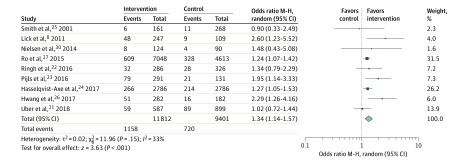
Association of Community Interventions With Survival Following Out-of-Hospital Cardiac Arrest M-H indicates Mantel-Haenszel.

**Figure 2.**
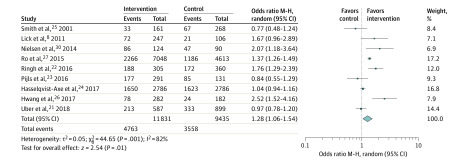
Association of Community Interventions With Bystander Cardiopulmonary Resuscitation Rate M-H indicates Mantel-Haenszel.

Nine studies (with a total of 21 266 patients experiencing OHCA) were included for the meta-analyses.^8,21,22,23,24,25,26,27,28^ The pooled estimates showed a significantly increased chance of survival to hospital discharge or 30 days’ survival (OR, 1.34; 95% CI, 1.14-1.57) with moderate heterogeneity (*I*^2^ = 33%; *P* = .15) (Figure 1). The pooled OR estimate of the bystander CPR rate was 1.28 (95% CI, 1.06-1.54) (Figure 2); however, there was substantial between-study heterogeneity (*I*^2^ = 82%; *P* < .001). When we removed studies that had substantially different designs, the effect size for survival appeared to increase and the heterogeneity reduced, although not consistently (eFigures 2-5 in the Supplement).

**Figure 3.**
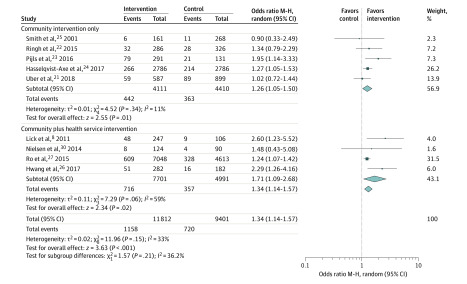
Forest Plot of Subgroup Comparison on Survival Following Out-of-Hospital Cardiac Arrest M-H indicates Mantel-Haenszel.

**Figure 4.**
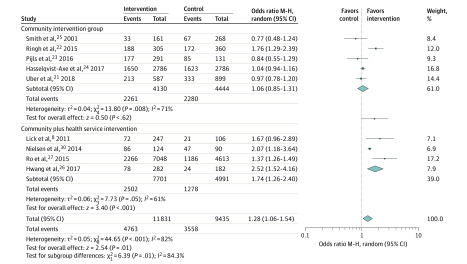
Forest Plot of Subgroup Comparison on Bystander Cardiopulmonary Resuscitation Rate M-H indicates Mantel-Haenszel.

Sensitivity analysis was conducted by omitting one study at a time. These analyses showed that omitting any 1 of 9 studies did not have a significant association with the original pooled ORs, with newly pooled ORs ranging from 1.23 (95% CI, 1.00-1.63; *I*^2^ = 87%) to 1.38 (95% CI, 1.10-1.73; *I*^2^ = 88%). The pooled model changing from random effects to fixed effects did not alter the significance. Furthermore, we explored whether there was a significant difference between community-only interventions and interventions with community and health service components on the study outcomes. Compared with community-only intervention, the combined community and health services intervention was not associated with a higher rate of survival (community plus intervention: OR, 1.71; 95% CI, 1.09-2.68 vs community alone: OR, 1.26; 95% CI, 1.05-1.50; *P* = .21) (Figure 3) but was associated with higher bystander CPR rates (community plus intervention: OR, 1.74; 95% CI, 1.26-2.40 vs community alone: OR, 10.6; 95% CI, 0.85-1.31; *P* = .01) (Figure 4). We also performed a restricted analysis that included only studies that targeted laypeople, and there still was an association with the interventions (eFigures 6 and 7 in the Supplement).

### Outcomes of Studies Not Included in Meta-analysis

Six studies were not included in the meta-analyses as they reported on observations of temporal changes in bystander resuscitation attempts and survival rates following OHCA during a period without clearly demonstrated interventions. We included these studies in our systematic review because they met our broad criteria of having a comparator; however, we did not include them in the meta-analysis because their design of examination of temporal trends was different from that of the other studies and were more prone to bias. These studies described the temporal trends in survival outcomes of OHCA after the implementation of national initiatives in Denmark, Singapore, and the US. In Denmark, there was a significant increase in bystander CPR from 21.1% (95% CI, 18.8%-23.4%) in 2001 to 44.9% (95% CI, 42.6%-47.1%) in 2010 (*P* < .001) and 30-day survival rates improved from 3.5% (95% CI, 2.5%-4.5%) in 2001 to 10.8% (95% CI, 9.4%-12.2%) in 2010 (*P* < .001).^29^ In Singapore, bystander CPR rates increased from 19.7% to 22.4% (*P* = .02) between 2001-2004 and 2010-2012, and the overall survival to discharge increased from 1.6% to 3.2% in the same period (adjusted OR, 2.2; 95% CI, 1.5-3.3).^31^ In the US, the HeartRescue Project was implemented in 5 states from 2011 to 2015. The authors observed modest temporal increases in bystander CPR rates (41.8%-43.5%; *P* < .001); however, no temporal changes were reported in survival following OHCA.^32^ The remaining 3 studies^15,30,33^ reported the results of statewide initiatives to improve bystander CPR and survival following OHCA in patients in North Carolina and Minnesota. The proportion of patients receiving bystander-initiated CPR increased significantly in both states, and improved survival was seen in North Carolina but not in Minnesota.

There was limited information on the cost of interventions in the included studies. In 2001, Smith et al^21^ had estimated the setup cost of training fire fighters and equipping their vehicles and fire stations with defibrillators and oxygen equipment to cover a metropolitan area in Australia of about 2 million people to be more than A$1.5 million and additionally over A$60 000 annually for maintenance of the consumables and devices and for refresher training. None of the other studies reported information on the costs and feasibility of implementing interventions.

### Risk of Bias Analysis

There was only 1 randomized clinical trial^23^ in this review and it was at low risk of bias according to the Cochrane Collaborations assessing tool for randomized clinical trials. The quality of the observational studies was evaluated by using the Newcastle-Ottawa Scale. Two studies were scored 7 stars and 12 studies were scored 5 or 6 stars. The main reasons for the loss of scores were lack of comparability of baseline characteristics between cohorts and selection of the nonexposed cohort from a different source.

## Discussion

In this systematic review and meta-analysis of a pooled 21 266 patients who experienced OHCA, better bystander CPR rate and survival rate were associated with implementation of community interventions. However, the quality of evidence was limited as comparators were nonrandomized in all but one study, and generalizability was limited as studies were mainly from high-income countries. There was moderate statistical heterogeneity among the 9 studies included in the meta-analysis regarding the survival rate of OHCA (*I*^2^ = 33%) and high heterogeneity among these articles when they were pooled for bystander CPR rate (*I*^2^ = 82%). Despite these heterogeneities, the results of sensitivity analyses were consistent and appeared to support the main result. We explored whether community intervention alone and community intervention combined with changes in health services had different outcomes. We found that the combined community and health services intervention was associated with a significantly higher rate of bystander CPR. A similar association was also observed with survival rate, although that finding was not statistically significant.

The analyses presented herein give some insights into the nature and potential novel components of community interventions that address first response to OHCA. New strategies, such as use of mobile communication devices, may improve outcomes as they may lead to earlier CPR. In the 4 studies reporting community-only interventions that used novel notification systems,^21,23,25,26^ trained volunteers were alerted by telephone, a text message, or a mobile positioning system to go to the cardiac arrest sites. A significant improvement in bystander CPR rates or survival to discharge or 30-day survival was achieved after these interventions. In the study conducted by Hasselqvist-Axe et al,^26^ notified first responders were first on the scene and initiated CPR before EMS personnel arrived in almost half of the OHCA cases. Similar findings have been reported in other programs using notification systems involving lay rescuers showing earlier defibrillation and an increase of OHCA survival rate.^34,35^ Technology and digital devices are promising intervention methods that can decrease bystander response time, but a key prerequisite of this strategy would be a sufficient number and distribution of trained lay volunteers. In contrast, nontargeted interventions may be less useful in improving bystander CPR or survival rate. In the study involving the training of laypersons conducted by Uber et al,^28^ 2235 nontargeted passersby were trained in 7 communities of Michigan with compression-only CPR, which is now a popular type of training method in community education.^36^ However, no improvement in bystander CPR or survival rates was seen, perhaps suggesting that the intensity of this intervention was inadequate or that nontargeted interventions are less effective. Previous systematic reviews have reported that training of targeted populations, such as family members of patients with cardiac disease,^37^ and certain communities with low bystander CPR rates, may be a useful way to improve bystander CPR rates and outcomes of OHCA.^10^

While the evidence synthesis in this review may contribute to a better understanding of the possibilities of community interventions, the findings suggest several challenges and barriers to implementing community interventions in large populations. Knowledge decay, panic, and lack of motivation are obstacles for laypeople in performing bystander CPR.^38,39,40^ There is evidence that only a third of trained laypersons performed CPR when they encountered a cardiac arrest situation.^41^ Studies of 1-time CPR training reported that adequate skills are retained only for 2 to 6 months after training.^42,43^ The relative infrequency of individuals performing CPR suggests that a greater prevalence of trained laypersons will be required to observe a significant increase in bystander CPR frequency.^44^

There are 4 factors that could be associated with the heterogeneity between studies: the definitions of bystanders, the criteria used to include OHCA population, the definition of survival outcomes, and the differences in educational level and health resources available in the countries in which the studies were conducted.

1. Bystander CPR: according to the updated Utstein criteria released by the International Liaison Committee on Resuscitation,^45^ bystander CPR refers to CPR performed by a person who is not responding to a cardiac arrest as part of an organized emergency medical system. In most of the studies, bystanders were laypersons, while in the studies of Pijl et al,^25^ Hasselqvist-Axe et al,^26^ and Smith et al,^21^ firefighters and policemen were included.
2. OHCA populations: subtle differences were observed in the selection of the OHCA populations. Six studies included OHCA cases with presumed cardiac origin,^8,22,23,24,25,27^ 1 study included all nontraumatic OHCA cases,^28^ and 2 studies included all-cause OHCA cases.^21,26^ Regarding the age of victims, 3 studies included only patients older than 18 years,^8,27,28^ 2 studies included patients older than 8 years,^23,26^ and the other 4 studies had no age limitations.^21,22,24,25^
3. Different definitions of survival following OHCA: 6 studies used survival to hospital discharge as the primary outcome of OHCA,^8,21,24,25,27,28^ and 3 studies reported 30-day survival.^22,23,26^
4. Variation in levels of intervention: there were differences in the level of public education and health resources available among the countries and regions in which the studies included in this review were conducted. Baseline bystander CPR rates were as high as 60% to 86% in some regions of Sweden and Denmark^22,23^ and lower than 30% in some other countries.^8,21,27^

These factors, as well as the variations between and within countries, in emergency medical systems, public educational level, government attention, and adequacy of funding for training need to be considered in the generalizability of these results as well as implementation of new programs.

### Limitations

This study has limitations. The main limitation of this study was the lack of randomized studies. In addition, there is a dearth of studies from diverse settings, including nonurban locations or low- and middle-income countries, a lack of data on costs and physical resources required for implementing community programs, and minimum information on participant and population details that may influence outcomes. Not all prospective studies that were included used active ascertainment, which is likely another source of heterogeneity. There was a practical challenge of interpreting the grouped results to inform clinical action given the wide spectrum of interventions grouped to generate the summary results. In addition, because the factors associated with outcomes of OHCA are multifaceted, it is possible that the survival improvement reported herein was confounded by temporal changes, concurrent interventions in EMS responses, and other undetected interventions.

## Conclusions

The results of this systematic review and meta-analysis suggest that community interventions are associated with higher survival rates following OHCA. Interventions that include both a community component and health service component appeared to be associated with improved bystander CPR greater than that of community-only intervention. Further research, particularly randomized clinical trials, is needed to understand whether community interventions to improve layperson CPR can improve outcomes in a diverse range of settings, whether certain approaches are more effective than others, the costs of implementation, and cost-effectiveness to aid further research translation.

## Notes

Supplement. **eMethods.** Search Strategy **eTable 1.** Other Related Outcomes Reported by Studies Included **eTable 2.** Interventions Reported in the Included Studies **eFigure 1.** The Study Selection Process **eFigure 2.** Forest Plot of Effects of Community Interventions on Survival From OHCA After Removing Nielsen 2014 and Ro 2015 **eFigure 3.** Forest Plot of Effects of Community Interventions on Bystander CPR After Removing Nielsen 2014 and Ro 2015 **eFigure 4.** Forest Plot of Subgroup Comparison on Survival From OHCA After Removing Nielsen 2014 and Ro 2015 **eFigure 5.** Forest Plot of Subgroup Comparison on Bystander CPR Rate After Removing Nielsen 2014 and Ro 2015 **eFigure 6.** Forest Plot of Effects of Community Interventions on Survival From OHCA After Removing Studies Involving Firefighters/Policemen (Smith 2001 and Hasselqvist-Axe 2017) **eFigure 7.** Forest Plot of Effects of Community Interventions on Bystander CPR After Removing Studies Involving Firefighters/Policemen (Smith 2001 and Hasselqvist-Axe 2017)
