# Supplementary material for: Assessment of Community Interventions for Bystander Cardiopulmonary Resuscitation in Out-of-Hospital Cardiac Arrest: A Systematic Review and Meta-analysis
Source: JAMA Netw Open. 2020 Jul 1;3(7):e209256. doi: 10.1001/jamanetworkopen.2020.9256 (PMC7330721; doi:10.1001/jamanetworkopen.2020.9256)
Supplement: Supplement. — eMethods. Search Strategy eTable 1. Other Related Outcomes Reported by Studies Included eTable 2. Interventions Reported in the Included Studies eFigure 1. The Study Selection Process eFigure 2. Forest Plot of Effects of Community Interventions on Survival From OHCA After Removing Nielsen 2014 and Ro 2015 eFigure 3. Forest Plot of Effects of Community Interventions on Bystander CPR After Removing Nielsen 2014 and Ro 2015 eFigure 4. Forest Plot of Subgroup Comparison on Survival From OHCA After Removing Nielsen 2014 and Ro 2015 eFigure 5. Forest Plot of Subgroup Comparison on Bystander CPR Rate After Removing Nielsen 2014 and Ro 2015 eFigure 6. Forest Plot of Effects of Community Interventions on Survival From OHCA After Removing Studies Involving Firefighters/Policemen (Smith 2001 and Hasselqvist-Axe 2017) eFigure 7. Forest Plot of Effects of Community Interventions on Bystander CPR After Removing Studies Involving Firefighters/Policemen (Smith 2001 and Hasselqvist-Axe 2017) [file jamanetwopen-3-e209256-s001.pdf]

Supplementary Online Content

Yu Y, Meng Q, Munot S, Nguyen TN, Redfern J, Chow CK. Assessment of community interventions for bystander cardiopulmonary resuscitation in out-of-hospital cardiac arrest: a systematic review and meta-analysis. *JAMA Netw Open*. 2020;3(7):e209256. doi:10.1001/jamanetworkopen.2020.9256

eMethods. Search Strategy

eTable 1. Other Related Outcomes Reported by Studies Included

eTable 2. Interventions Reported in the Included Studies

eFigure 1. The Study Selection Process

eFigure 2. Forest Plot of Effects of Community Interventions on Survival From OHCA After Removing Nielsen 2014 and Ro 2015

eFigure 3. Forest Plot of Effects of Community Interventions on Bystander CPR After Removing Nielsen 2014 and Ro 2015

eFigure 4. Forest Plot of Subgroup Comparison on Survival From OHCA After Removing Nielsen 2014 and Ro 2015

eFigure 5. Forest Plot of Subgroup Comparison on Bystander CPR Rate After Removing Nielsen 2014 and Ro 2015

eFigure 6. Forest Plot of Effects of Community Interventions on Survival From OHCA After Removing Studies Involving Firefighters/Policemen (Smith 2001 and Hasselqvist-Axe 2017)

eFigure 7. Forest Plot of Effects of Community Interventions on Bystander CPR After Removing Studies Involving Firefighters/Policemen (Smith 2001 and Hasselqvist-Axe 2017)

This supplementary material has been provided by the authors to give readers additional information about their work.

© 2020 Yu Y et al. *JAMA Network Open.*

eMethods. Search Strategy

1. Cardiopulmonary Resuscitation/

2. Cardiopulmonary Resuscitation/ed [Education]

3. exp Resuscitation/

4. Resuscitation/ed [Education]

5. Cardio-pulmonary resuscitation.mp.

6. CPR certification.mp.

7. CPR training.mp.

8. 1 or 2 or 3 or 4 or 5 or 6 or 7

9. exp First Aid/

10. First Aid/ed [Education]

11. Basic life support.mp.

12. BLS training.mp.

13. 9 or 10 or 11 or 12

14. (lay person* or lay responder* or layperson* or bystander* or layman or laywoman or laymen or laywomen or first responder*).mp.

15. 13 or 14

16. exp Education, Nonprofessional/

17. exp Education/

18. exp Health Education/

19. Teaching/

20. Learning/

21. 16 or 17 or 18 or 19 or 20

22. 8 and 15 and 21

23. ((CPR or cardiopulmonary resuscitation or BLS or basic life support or resuscitation) adj3 (train* or teach* or educat* or learn*)).mp.

24. cardiac arrest.mp.

25. 14 and 21 and 24

26. 22 or 23 or 25

27. (survival or cerebral performance category or ROSC or return of spontaneous circulation).mp.

28. 26 and 27

© 2020 Yu Y et al. *JAMA Network Open.*

eTable 1. Other related outcomes reported by studies included

Author, Year	Number of people with OHCA	Bystander witnessed OHCA (%)	Bystander defibrillation (%)	ROSC (%)	Admitted to hospital (%)	Good neurological outcome (%)
Smith, et al. 2001	Control : 268<br>Intervention: 161	Control : 78 (29.1%)<br>Intervention: 35 (21.7%)<br>No p value and OR	NA	NA	NA	NA
Lick, et al. 2011	Control: 106<br>Intervention: 247	NA	NA	Control: 40 (38%)<br>Intervention: 116 (47%)<br>OR 1.46, [95% CI 0.90–2.40], p=0.13	Control: 37 (35%)<br>Intervention: 95 (38%)<br>OR 1.17, [95% CI 0.71–1.93], p=0.55	NA
Wissenberg, et al. 2013	2001: 1262<br>2010: 1906	2001: 599 (51.6%)<br>2010: 1020 (53.9%)<br>p=0.001, no OR	2001: 13 (1.1%)<br>2010: 36 (2.2%)<br>p=0.003, no OR	NA	2001: 91 (7.9%)<br>2010: 354 (21.8%)<br>p<0.001, no OR	NA
Nielsen, et al. 2014	Control: 90<br>Intervention: 124	Control: 35 (39%)<br>Intervention: 49 (40%)<br>No p value and OR	NA	Control: 17 (19%)<br>Intervention: 28 (23%)<br>p=0.61, no OR	NA	NA
Ringh, et al. 2015	Control: 361<br>Intervention: 306	Control: 186 (57.6%)<br>Intervention: 165 (56.9%)<br>No p value and OR	NA	Control: 105(29.1%)<br>Intervention: 90 (29.4%)<br>Difference 0.3, [95% CI −6.5–7.3], p=0.93	NA	NA
Lai, et al. 2015	Before: 2428<br>After: 3025	Before: 1318 (54.3%)<br>After: 1483 (49.0%)<br>No p value and OR	Before: 0 (0.0%)<br>After: 29 (1.0%)<br>No p value and OR	Before: 239 (15.0%)<br>After: 526 (23.2%)<br>OR 1.7, [95% CI 1.4–2.0], no p value	Before: 111 (6.9%)<br>After: 323 (14.3%)<br>OR 2.2, [95% CI 1.8–2.8], no p value	NA

© 2020 Yu Y et al. *JAMA Network Open.*

eTable 1. Other related outcomes reported by studies included (continued)

Author, Year	Number of people with OHCA	Bystander witnessed OHCA (%)	Bystander defibrillation (%)	ROSC (%)	Admitted to hospital (%)	Good neurological outcome (%)
Hansen, et al. 2015	2010: 1167<br>2013: 1341	2010: 502 (43.0%)<br>2013: 620 (46.2%)<br>p=0.22, no OR	2010: 34 (9.3%)<br>2013: 127 (6.0%)<br>p=0.16, no OR	2010: 285 (24.5%)<br>2013: 406 (30.3%)<br>p<0.01, no OR	2010: 263 (22.5%)<br>2013: 366 (27.3%)<br>p<0.01, no OR	2010: 82 (7.1%)<br>2013: 129 (9.7%)<br>p=0.02, no OR
Ro, et al. 2015	Seoul Control: 1054<br>Intervention: 2355<br><br>Osaka Control: 3559<br>Intervention: 4693	Seoul Control: 620 (58.8%)<br>Intervention: 1201 (51.0%)<br>p<0.01, no OR<br><br>Osaka Control: 1470 (41.3%)<br>Intervention: 1882(40.1%)<br>p=0.30, no OR	Seoul Control: 0 (0%)<br>Intervention: 0 (0%)<br>p=0.2, no OR<br>Osaka Control: 14 (0.4%)<br>Intervention: 47 (1.0%)<br>p<0.01, no OR	NA	NA	Seoul Control: 15 (1.4%)<br>Intervention: 94 (4.0%)<br>p<0.01, no OR<br><br>Osaka Control: 127 (3.6%)<br>Intervention: 224 (4.8%)<br>p=0.30, no OR
(Pijls, et al. 2016	Control: 131<br>Intervention: 291	Control: 99 (75.6%)<br>Intervention: 218 (74.9%)<br>p=0.885, no OR	NA	Control: 42 (32.3%)<br>Intervention: 121 (41.7%) p=0.098, no OR	NA	NA
Hasselqvist, et al. 2017	Control: 5155<br>Intervention:3543	Control: 3062 (60.5%)<br>Intervention: 2001 (58.3%)<br>No p value and OR	Control: 94 (1.8%)<br>Intervention: 65 (1.8%)<br>No p value and OR	NA	Control: 1143 (32.4%)<br>Intervention: 1143 (32.4%)<br>No p value and OR	NA
Hwang, et al. 2017	Control: 182<br>Intervention:282	Control: 109 (59.9%)<br>Intervention: 172 (61.0%)<br>p=0.843, no OR	Control: 0 (0%)<br>Intervention: 1 (0.3%)<br>No p value and OR	Control: 36 (19.8%)<br>Intervention: 125 (44.3%)<br>No p value and OR	Control: 29 (15.9%)<br>Intervention: 101 (35.8%)<br>p<0.001, no OR	Control: 6 (3.3%)<br>Intervention: 24 (8.5%)<br>No p value and OR
Diepen, et al. 2017	2011: 6,762<br>2015: 16,103	2011: 2673 (39.5%)<br>2015: 6609 (41.0%)<br>p=0.001, no OR	2011: 217 (3.2%)<br>2015: 902 (5.6%)<br>p<0.001, no OR	NA	2011: 2712 (40.1%)<br>2015: 5475 (34.0%)<br>p<0.001, no OR	2011: 701 (10.4%)<br>2015: 1426 (8.9%)<br>p=0.002, no OR

© 2020 Yu Y et al. *JAMA Network Open.*

eTable 1. Other related outcomes reported by studies included (continued)

Author, Year	Number of people with OHCA	Bystander witnessed OHCA (%)	Bystander defibrillation (%)	ROSC (%)	Admitted to hospital (%)	Good neurological outcome (%)
Fordyce, et al. 2017	At Home<br>2010: 1063<br>2014: 1242<br><br>In Public:<br>2010: 470<br>2014: 605	At Home<br>2010: 425 (40.0%)<br>2014: 532 (42.8%)<br>p=0.13, no OR<br>In Public:<br>2010: 213 (45.3%)<br>2014: 306 (50.6%)<br>p=0.22, no OR	At Home<br>2010: 5 (1.6%)<br>2014: 2 (0.5%)<br>p=0.06, no OR<br>In Public:<br>2010: 37 (21.1%)<br>2014: 49 (19.9%)<br>p=0.19, no OR	NA	NA	At Home<br>2010: 52 (4.9%)<br>2014: 76 (6.1%)<br>p=0.06, no OR<br>In Public:<br>2010: 44 (9.5%)<br>2014: 89 (14.7%)<br>p=0.02, no OR
Adabag, et al. 2017	2011: 1067<br>2014: 1473	2011: 442 (41%)<br>2014: 661 (45%)<br>p=0.06, no OR	NA	NA	2011: 343 (32%)<br>2014: 405 (27%)<br>p=0.004, no OR	2011: 150 (14%)<br>2014: 157 (11%)<br>p=0.02, no OR
Uber, et al. 2018	Control: 899<br>Intervention: 587	Control: 414 (46%)<br>Intervention 232 (40%)<br>p=0.01, no OR	NA	Control: 263 (29%)<br>Intervention: 181 (31%)<br>p=0.52, no OR	NA	Control: 75 (8%)<br>Intervention: 51 (9%)<br>p=0.87, no OR

OHCA: Out-of-hospital cardiac arrest; ROSC: Return of spontaneous circulation; OR: odds ratio.

© 2020 Yu Y et al. *JAMA Network Open.*

eTable 2. Interventions reported in the included studies

Author Year	Interventions
Smith, et al. 2001	**Community intervention**<br>Additional dispatch of fire fighters (trained in BLS, use of AEDs in an 8-day training course and equipped with AEDs) to the existing two-tiered EMS dispatch system
Ringh, et al. 2015	**Community intervention**<br>Mobile phone dispatch of laypersons for CPR
Pijls, et al. 2016	**Community intervention**<br>Text message alert system to activate trained volunteers
Hasselqvist, et al. 2017	**Community intervention**<br>EMS dispatch of first responders for CPR
Uber, et al. 2018	**Community intervention**<br>One time, point-of-contact compression-only CPR training at public locations on a single day
Lick, et al. 2011	**Community Intervention**<br>- increasing public awareness, community CPR training<br>- distribution of AHA CPR Anytime 25-min training kit to school-students and their family members<br>- CPR training was provided to civic groups, city employees, and various businesses<br>- public television produced and broadcasted a 30-min documentary on CPR education program<br>- additional deployment of AEDs in schools and public places<br>**Health services intervention**<br>- retraining of all EMS personnel in high performance CPR<br>- protocols for therapeutic hypothermia, coronary artery evaluation.
Wissenberg, et al. 2013	**Community intervention**<br>- implementation of mandatory resuscitation training in elementary schools and in those acquiring a driver's license<br>- increasing voluntary first aid training<br>- distribution of CPR self-instruction training kits<br>- increasing AEDs located outside hospitals<br>**Health services intervention**<br>- nationwide improvement of DACPR<br>- efforts to improve therapeutic hypothermia and early revascularization<br>- overall strengthening of EMS system.
Nielsen, et al. 2014	**Community intervention**<br>- Mass media campaign & widespread education (including broadcasting resuscitation on local television)<br>- Distribution of 24-min DVD-based-self-instruction BLS courses; 4-h BLS/AED courses for the public.<br>- Increase of AEDs in public area<br>**Health services intervention**<br>- Training staff at the hospitals and EMS.
Lai, et al. 2015	**Community intervention**<br>- Public education on CPR<br>- Public access defibrillators<br>**Health services intervention**<br>- Increase in the number of ambulances<br>- Improvement of paramedic skills<br>- Introduction of therapeutic hypothermia.

© 2020 Yu Y et al. *JAMA Network Open.*

eTable 2. Interventions reported in the included studies (continued)

Author Year	Interventions
Hansen, et al. 2015	**Community intervention**<br>- Statewide initiatives: training the population in CPR and AED use.<br>- training first responders in team-based CPR including AED use and high-performance CPR<br>**Health services intervention**<br>- Training dispatch centres in recognizing cardiac arrest symptoms in order to implement bystander CPR within 60 seconds of call receipt.
Ro, et al. 2015	**Community intervention**<br>- Public education and CPR training programmes<br>- high-quality bystander CPR education programmes<br>- Increasing AEDs in public locations<br>- Enactment of the Good Samaritan Law<br>**Health services intervention**<br>- EMS system quality control programmes<br>- advanced CPR skill training for EMS staff
Hwang, et al. 2017	**Community intervention**<br>- public CPR training, teaching compression-only CPR in addition to standard BLS courses<br>**Health services intervention**<br>- implementation of DACPR protocol<br>- quality improvement of EMS<br>- advanced life support protocol in hospital
Diepen, et al. 2017	**Community intervention:**<br>Arizona SHARE Bystander CPR Initiative (brief online video training; free training kits sent to school students; free training in many locations throughout the state, etc.); Minnesota Bystander CPR Training, etc.<br>**Health services intervention**<br>- Heart Rescue Project (establishment of a statewide resuscitation registry,<br>- measure performance and outcomes and a focus on incremental system quality improvement
Fordyce, et al. 2017	The North Carolina RACE CARS program<br>**Community intervention**<br>- community training in chest compression–only CPR<br>- first-responder training in team-based CPR<br>**Health services intervention**<br>- providing emergency medical dispatcher training on early recognition of cardiac arrest and instructing bystanders to provide CPR,<br>- several in-hospital interventions
Adabag, et al. 2017	State-wide integrated resuscitation program<br>**Community intervention**<br>- training sessions for CPR and AED use in schools, workplaces and special events<br>**Health services intervention**<br>- increasing the utilization of therapeutic hypothermia, emergency cardiac catheterization<br>- gathering first responders, EMS, police and fire departments, hospital emergency departments, cardiology, intensive care unit services under the same organization

Abbreviations: BLS, basic life support; AED, automatic external defibrillator; EMS, emergency medical services; CPR, cardiopulmonary resuscitation; AHA, American heart association; DACPR, dispatcher-guided bystander CPR; RACE CARS: regional approach to cardiovascular emergencies cardiac arrest resuscitation.

© 2020 Yu Y et al. *JAMA Network Open.*

eFigure 1. The study selection process

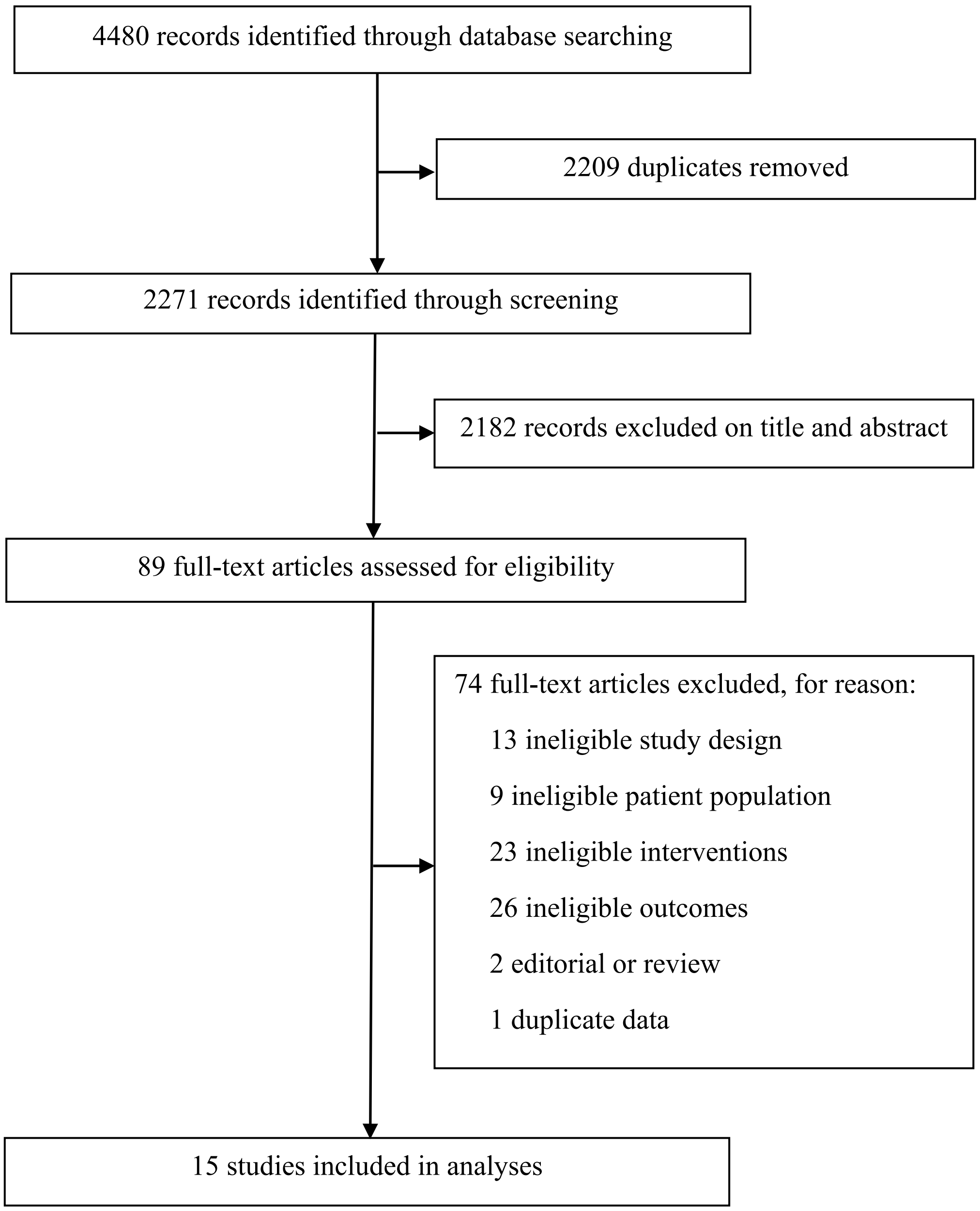

© 2020 Yu Y et al. *JAMA Network Open.*

eFigure 2. Forest plot of effects of community interventions on survival from OHCA after removing Nielsen 2014 and Ro 2015

	Intervention		Control			Odds Ratio	
Study or Subgroup	Events	Total	Events	Total	Weight	M-H, Random, 95% CI	Year
Smith 2001	6	161	11	268	5.0%	0.90 [0.33, 2.49]	2001
Lick 2011	48	247	9	106	8.1%	2.60 [1.23, 5.52]	2011
Nielsen 2014	8	124	4	90	0.0%	1.48 [0.43, 5.08]	2014
Ro 2015	609	7048	328	4613	0.0%	1.24 [1.07, 1.42]	2015
Ringh 2015	32	286	28	326	13.1%	1.34 [0.79, 2.29]	2015
Pijls 2016	79	291	21	131	13.1%	1.95 [1.14, 3.33]	2016
Hasselqvist-Axe 2017	266	2786	214	2786	28.8%	1.27 [1.05, 1.53]	2017
Hwang 2017	51	282	16	182	11.4%	2.29 [1.26, 4.16]	2017
Uber 2018	59	587	89	899	20.5%	1.02 [0.72, 1.44]	2018
**Total (95% CI)**		**4640**		**4698**	**100.0%**	**1.44 [1.13, 1.84]**	
Total events	541		388				

Heterogeneity: Tau² = 0.05; Chi² = 11.35, df = 6 (P = 0.08); I² = 47%

Test for overall effect: Z = 2.92 (P = 0.004)

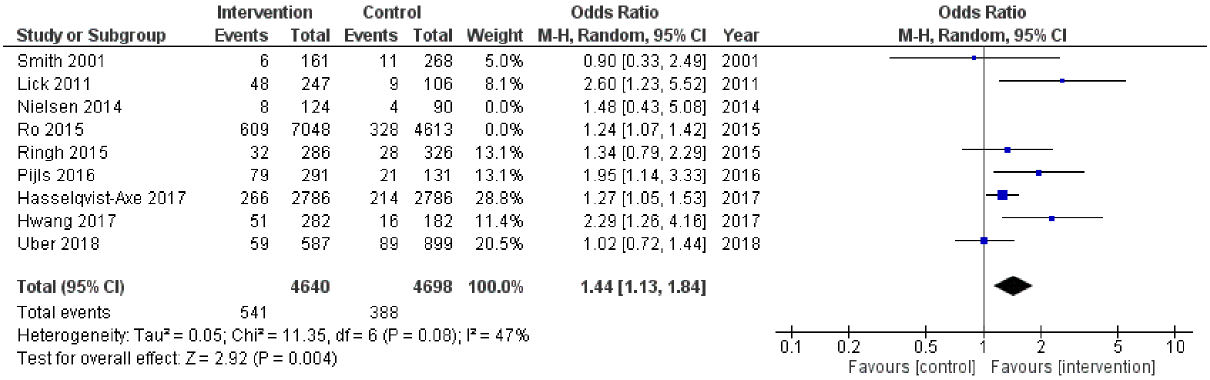

© 2020 Yu Y et al. *JAMA Network Open.*

eFigure 3. Forest plot of effects of community interventions on bystander CPR after removing Nielsen 2014 and Ro 2015

	Intervention		Control			Odds Ratio	
Study or Subgroup	Events	Total	Events	Total	Weight	M-H, Random, 95% CI	Year
Smith 2001	33	161	67	268	11.8%	0.77 [0.48, 1.24]	2001
Lick 2011	72	247	21	106	10.2%	1.67 [0.96, 2.89]	2011
Nielsen 2014	86	124	47	90	0.0%	2.07 [1.18, 3.64]	2014
Ro 2015	2266	7048	1186	4613	0.0%	1.37 [1.26, 1.49]	2015
Ringh 2015	188	305	172	360	15.7%	1.76 [1.29, 2.39]	2015
Pijls 2016	177	291	85	131	12.8%	0.84 [0.55, 1.29]	2016
Hasselqvist-Axe 2017	1650	2786	1623	2786	20.3%	1.04 [0.94, 1.16]	2017
Hwang 2017	78	282	24	182	11.1%	2.52 [1.52, 4.16]	2017
Uber 2018	213	587	333	899	18.1%	0.97 [0.78, 1.20]	2018
**Total (95% CI)**		**4659**		**4732**	**100.0%**	**1.21 [0.95, 1.55]**	
Total events	2411		2325				

Heterogeneity: Tau² = 0.07; Chi² = 27.37, df = 6 (P = 0.0001); I² = 78%
Test for overall effect: Z = 1.55 (P = 0.12)

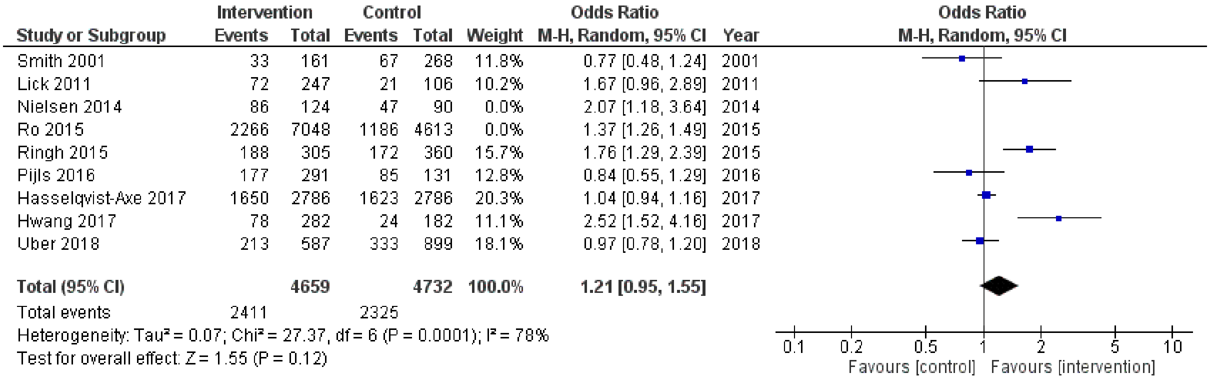

© 2020 Yu Y et al. *JAMA Network Open.*

eFigure 4. Forest plot of subgroup comparison on survival from OHCA after removing Nielsen 2014 and Ro 2015

Study or Subgroup	Intervention Events	Intervention Total	Control Events	Control Total	Weight	Odds Ratio M-H, Random, 95% CI	Year
**1.2.1 Community intervention only**							
Smith 2001	6	161	11	268	5.0%	0.90 [0.33, 2.49]	2001
Ringh 2015	32	286	28	326	13.1%	1.34 [0.79, 2.29]	2015
Pijls 2016	79	291	21	131	13.1%	1.95 [1.14, 3.33]	2016
Hasselqvist-Axe 2017	266	2786	214	2786	28.8%	1.27 [1.05, 1.53]	2017
Uber 2018	59	587	89	899	20.5%	1.02 [0.72, 1.44]	2018
**Subtotal (95% CI)**		**4111**		**4410**	**80.5%**	**1.26 [1.05, 1.50]**	
Total events	442		363				
Heterogeneity: Tau² = 0.01; Chi² = 4.52, df = 4 (P = 0.34); I² = 11%							
Test for overall effect: Z = 2.55 (P = 0.01)							
**1.2.2 Community plus health service intervention**							
Lick 2011	48	247	9	106	8.1%	2.60 [1.23, 5.52]	2011
Nielsen 2014	8	124	4	90	0.0%	1.48 [0.43, 5.08]	2014
Ro 2015	609	7048	328	4613	0.0%	1.24 [1.07, 1.42]	2015
Hwang 2017	51	282	16	182	11.4%	2.29 [1.26, 4.16]	2017
**Subtotal (95% CI)**		**529**		**288**	**19.5%**	**2.41 [1.51, 3.84]**	
Total events	99		25				
Heterogeneity: Tau² = 0.00; Chi² = 0.07, df = 1 (P = 0.80); I² = 0%							
Test for overall effect: Z = 3.68 (P = 0.0002)							
**Total (95% CI)**		**4640**		**4698**	**100.0%**	**1.44 [1.13, 1.84]**	
Total events	541		388				
Heterogeneity: Tau² = 0.05; Chi² = 11.35, df = 6 (P = 0.08); I² = 47%							
Test for overall effect: Z = 2.92 (P = 0.004)							
Test for subgroup differences: Chi² = 6.49, df = 1 (P = 0.01), I² = 84.6%							

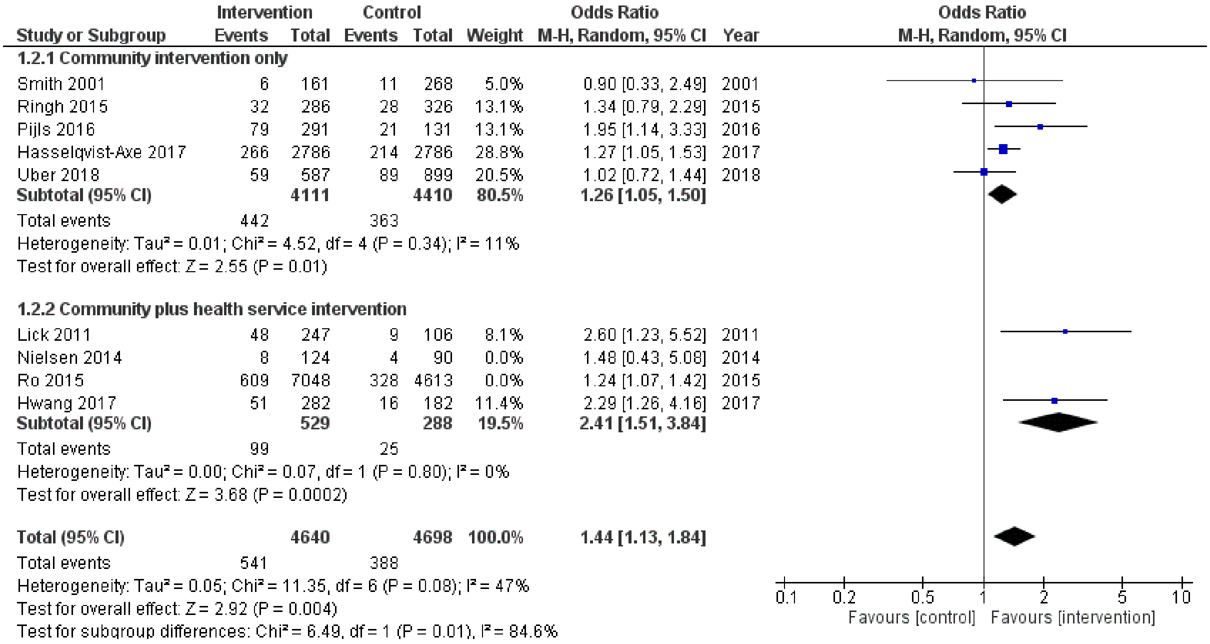

© 2020 Yu Y et al. *JAMA Network Open.*

eFigure 5. Forest plot of subgroup comparison on bystander CPR rate after removing Nielsen 2014 and Ro 2015

	Intervention		Control			Odds Ratio	
Study or Subgroup	Events	Total	Events	Total	Weight	M-H, Random, 95% CI	Year
**1.1.1 Community intervention only**							
Smith 2001	33	161	67	268	11.8%	0.77 [0.48, 1.24]	2001
Ringh 2015	188	305	172	360	15.7%	1.76 [1.29, 2.39]	2015
Pijls 2016	177	291	85	131	12.8%	0.84 [0.55, 1.29]	2016
Hasselqvist-Axe 2017	1650	2786	1623	2786	20.3%	1.04 [0.94, 1.16]	2017
Uber 2018	213	587	333	899	18.1%	0.97 [0.78, 1.20]	2018
**Subtotal (95% CI)**		**4130**		**4444**	**78.7%**	**1.06 [0.85, 1.31]**	
Total events	2261		2280				
Heterogeneity: Tau² = 0.04; Chi² = 13.80, df = 4 (P = 0.008); I² = 71%							
Test for overall effect: Z = 0.50 (P = 0.62)							
**1.1.2 Community plus health service intervention**							
Lick 2011	72	247	21	106	10.2%	1.67 [0.96, 2.89]	2011
Nielsen 2014	86	124	47	90	0.0%	2.07 [1.18, 3.64]	2014
Ro 2015	2266	7048	1186	4613	0.0%	1.37 [1.26, 1.49]	2015
Hwang 2017	78	282	24	182	11.1%	2.52 [1.52, 4.16]	2017
**Subtotal (95% CI)**		**529**		**288**	**21.3%**	**2.08 [1.39, 3.12]**	
Total events	150		45				
Heterogeneity: Tau² = 0.01; Chi² = 1.18, df = 1 (P = 0.28); I² = 15%							
Test for overall effect: Z = 3.56 (P = 0.0004)							
**Total (95% CI)**		**4659**		**4732**	**100.0%**	**1.21 [0.95, 1.55]**	
Total events	2411		2325				
Heterogeneity: Tau² = 0.07; Chi² = 27.37, df = 6 (P = 0.0001); I² = 78%							
Test for overall effect: Z = 1.55 (P = 0.12)							
Test for subgroup differences: Chi² = 8.37, df = 1 (P = 0.004), I² = 88.1%							

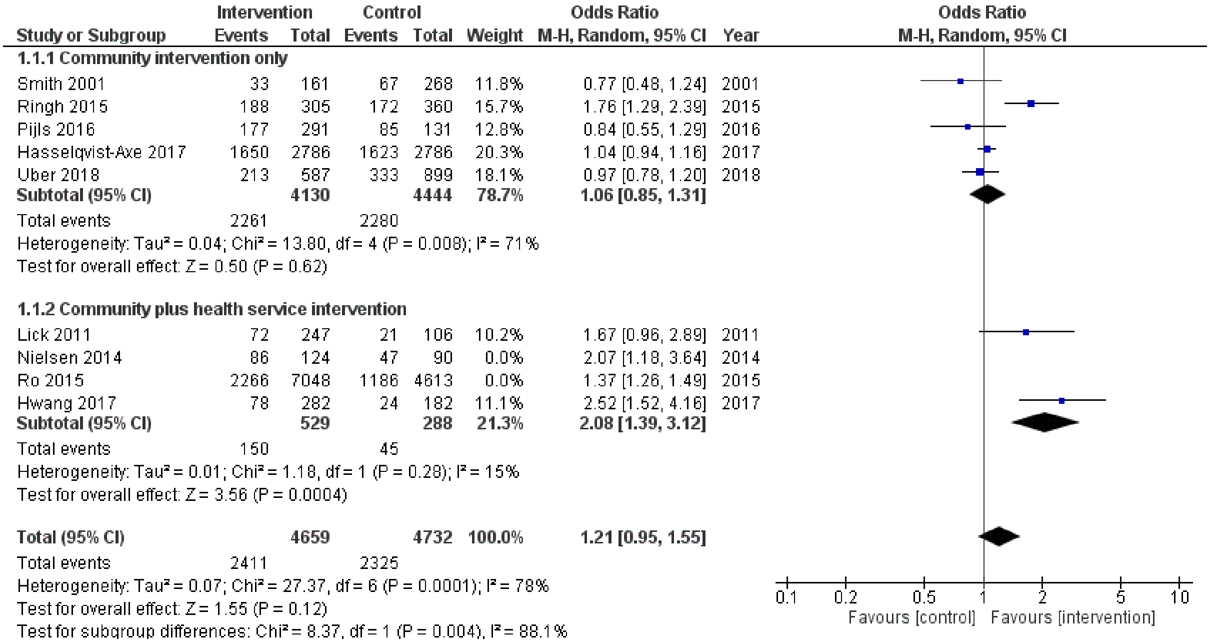

© 2020 Yu Y et al. *JAMA Network Open.*

eFigure 6. Forest plot of effects of community interventions on survival from OHCA after removing studies involving firefighters/policemen (Smith 2001 and Hasselqvist-Axe 2017):

	Intervention		Control			Odds Ratio	
Study or Subgroup	Events	Total	Events	Total	Weight	M-H, Random, 95% CI	Year
Smith 2001	6	161	11	268	0.0%	0.90 [0.33, 2.49]	2001
Lick 2011	48	247	9	106	7.9%	2.60 [1.23, 5.52]	2011
Nielsen 2014	8	124	4	90	3.4%	1.48 [0.43, 5.08]	2014
Ro 2015	609	7048	328	4613	31.6%	1.24 [1.07, 1.42]	2015
Ringh 2015	32	286	28	326	12.8%	1.34 [0.79, 2.29]	2015
Pijls 2016	79	291	21	131	12.8%	1.95 [1.14, 3.33]	2016
Hasselqvist-Axe 2017	266	2786	214	2786	0.0%	1.27 [1.05, 1.53]	2017
Hwang 2017	51	282	16	182	11.1%	2.29 [1.26, 4.16]	2017
Uber 2018	59	587	89	899	20.3%	1.02 [0.72, 1.44]	2018
**Total (95% CI)**		**8865**		**6347**	**100.0%**	**1.45 [1.14, 1.85]**	
Total events	886		495				

Heterogeneity: Tau² = 0.04; Chi² = 11.47, df = 6 (P = 0.08); I² = 48%

Test for overall effect: Z = 3.07 (P = 0.002)

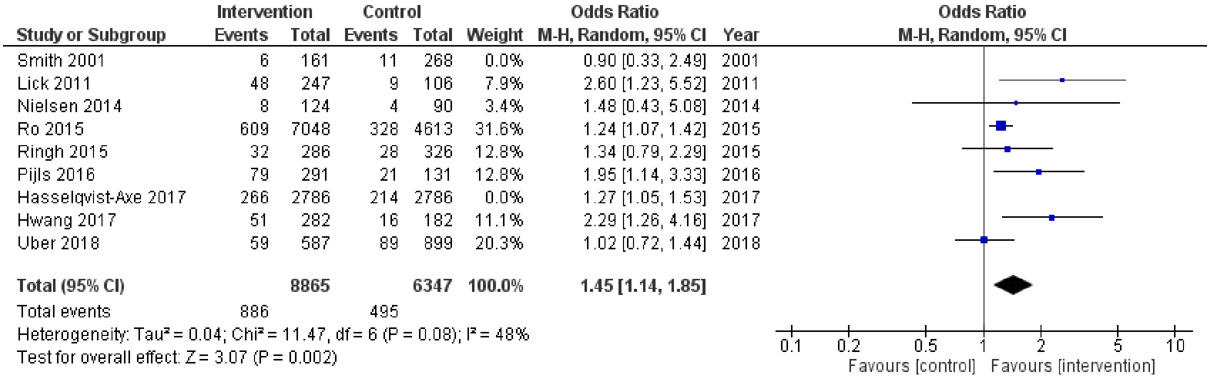

© 2020 Yu Y et al. *JAMA Network Open.*

eFigure 7. Forest plot of effects of community interventions on bystander CPR after removing studies involving firefighters/policemen (Smith 2001 and Hasselqvist-Axe 2017):

	Intervention		Control			Odds Ratio	
Study or Subgroup	Events	Total	Events	Total	Weight	M-H, Random, 95% CI	Year
Smith 2001	33	161	67	268	0.0%	0.77 [0.48, 1.24]	2001
Lick 2011	72	247	21	106	9.9%	1.67 [0.96, 2.89]	2011
Nielsen 2014	86	124	47	90	9.7%	2.07 [1.18, 3.64]	2014
Ro 2015	2266	7048	1186	4613	21.8%	1.37 [1.26, 1.49]	2015
Ringh 2015	188	305	172	360	16.0%	1.76 [1.29, 2.39]	2015
Pijls 2016	177	291	85	131	12.7%	0.84 [0.55, 1.29]	2016
Hasselqvist-Axe 2017	1650	2786	1623	2786	0.0%	1.04 [0.94, 1.16]	2017
Hwang 2017	78	282	24	182	11.0%	2.52 [1.52, 4.16]	2017
Uber 2018	213	587	333	899	18.8%	0.97 [0.78, 1.20]	2018
**Total (95% CI)**		**8884**		**6381**	**100.0%**	**1.42 [1.13, 1.80]**	
Total events	3080		1868				

Heterogeneity: Tau² = 0.06; Chi² = 25.37, df = 6 (P = 0.0003); I² = 76%
Test for overall effect: Z = 2.98 (P = 0.003)

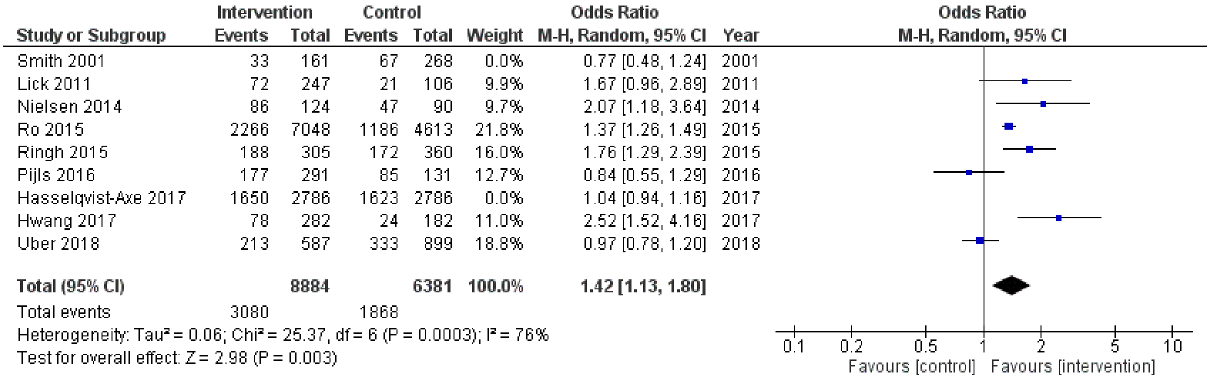

© 2020 Yu Y et al. *JAMA Network Open.*
